# 8-Hy­droxy-2-methyl­quinolinium tetra­chlorido(quinolin-8-olato-κ^2^ *N*,*O*)stan­nate(IV)

**DOI:** 10.1107/S1600536812019459

**Published:** 2012-05-05

**Authors:** Ezzatollah Najafi, Mostafa M. Amini, Seik Weng Ng

**Affiliations:** a Department of Chemistry, General Campus, Shahid Beheshti University, Tehran 1983963113, Iran; b Department of Chemistry, University of Malaya, 50603 Kuala Lumpur, Malaysia; c Chemistry Department, Faculty of Science, King Abdulaziz University, PO Box 80203 Jeddah, Saudi Arabia

## Abstract

The reaction of 8-hy­droxy­quinoline, 2-methyl­quinolin-8-ol and stannic chloride yields the protonated 8-hy­droxy-2-methyl­quinolinium species. In the title salt, (C_10_H_10_NO)[Sn(C_9_H_6_NO)Cl_4_], the Sn^IV^ cation is *N*,*O*-chelated by the quinolin-8-olate anion and is further coordinated by four Cl^−^ anions in a distorted *cis*-SnNOCl_4_ octa­hedral geometry. In the crystal, the cation is linked to the anion by an O—H⋯O hydrogen bond.

## Related literature

For the methanol solvate of the salt, see: Najafi *et al.* (2011 ▶).
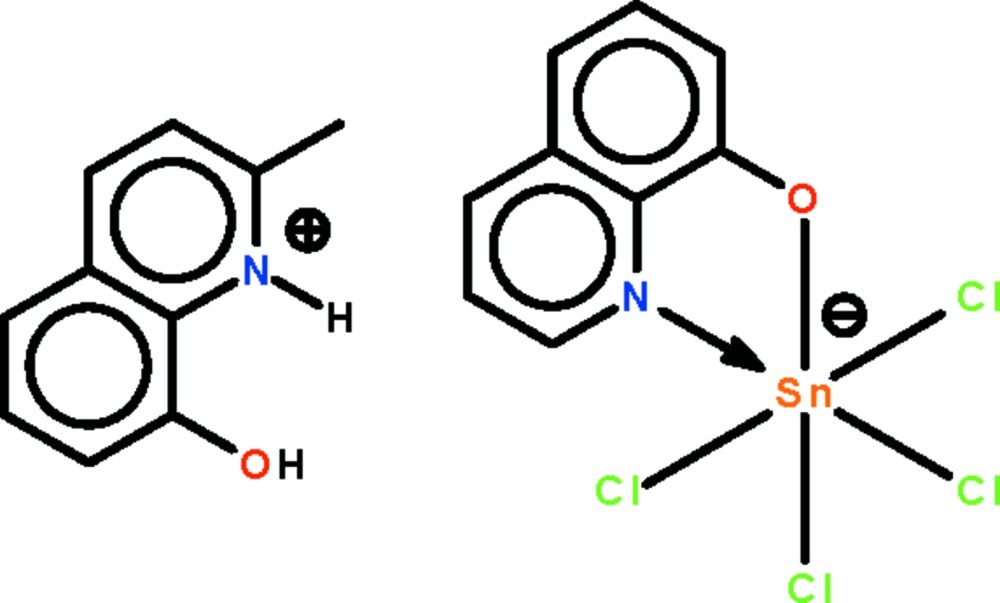


## Experimental

### 

#### Crystal data

(C_10_H_10_NO)[Sn(C_9_H_6_NO)Cl_4_]
*M* *_r_* = 564.83
Monoclinic, 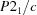

*a* = 8.9431 (3) Å
*b* = 11.5892 (4) Å
*c* = 20.1795 (8) Å
β = 101.347 (4)°
*V* = 2050.59 (13) Å^3^
*Z* = 4
Mo *K*α radiation
μ = 1.79 mm^−1^
*T* = 100 K
0.30 × 0.25 × 0.20 mm

#### Data collection

Agilent SuperNova Dual diffractometer with an Atlas detector
Absorption correction: multi-scan (*CrysAlis PRO*; Agilent, 2012 ▶) *T*_min_ = 0.616, *T*_max_ = 0.717
13674 measured reflections
4723 independent reflections
4222 reflections with *I* > 2σ(*I*)
*R*_int_ = 0.029

#### Refinement

*R*[*F* ^2^ > 2σ(*F* ^2^)] = 0.025
*wR*(*F* ^2^) = 0.055
*S* = 1.06
4723 reflections
262 parameters
2 restraints
H atoms treated by a mixture of independent and constrained refinement
Δρ_max_ = 0.48 e Å^−3^
Δρ_min_ = −0.54 e Å^−3^

### 

Data collection: *CrysAlis PRO* (Agilent, 2012 ▶); cell refinement: *CrysAlis PRO*; data reduction: *CrysAlis PRO*; program(s) used to solve structure: *SHELXS97* (Sheldrick, 2008 ▶); program(s) used to refine structure: *SHELXL97* (Sheldrick, 2008 ▶); molecular graphics: *X-SEED* (Barbour, 2001 ▶); software used to prepare material for publication: *publCIF* (Westrip, 2010 ▶).

## Supplementary Material

Crystal structure: contains datablock(s) global, I. DOI: 10.1107/S1600536812019459/xu5531sup1.cif

Structure factors: contains datablock(s) I. DOI: 10.1107/S1600536812019459/xu5531Isup2.hkl

Additional supplementary materials:  crystallographic information; 3D view; checkCIF report

## Figures and Tables

**Table 1:** Hydrogen-bond geometry (Å, °)

*D*—H⋯*A*	*D*—H	H⋯*A*	*D*⋯*A*	*D*—H⋯*A*
O2—H1⋯O1	0.84 (1)	1.86 (1)	2.683 (2)	168 (3)

**Fig. 1.**
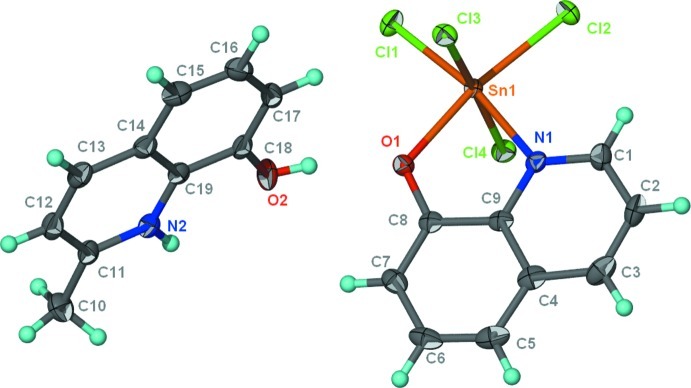
Thermal ellipsoid plot (Barbour, 2001) of (C10H10NO)[SnCl4(C9H6NO)] at the 70% probability level; hydrogen atoms are drawn as spheres of arbitrary radius.

## supplementary crystallographic information

### Comment

The reaction of 8-hydroxyquinoline, 2-methyl-8-hydroxyquinoline and stannic chloride in methanol yields a protonated 2-methyl-8-hydroxyquinoline species. The Sn^IV^atom in the methanol-solvated salt, (C_10_H_10_NO)[SnCl_4_(C_9_H_6_NO)]^.^2CH_3_OH, is *N*,*O*-chelated by the quinolin-8-olate (Najafi *et al.*, 2011). A repeat of the synthesis but isopropyl alcohol in place of methanol yielded the unsolvated salt (Scheme I). The Sn^IV^atom in the salt is *N*,*O*-chelated by the quinolin-8-olate in a *cis*-SnNOCl_4_ octahedral geometry. The cation is linked to the anion by an O–H···O hydrogen bond (Fig. 1, Table 1).

### Experimental

Stannic chloride pentahydrate (0.35 g, 1 mmol), 8-hydroxyquinoline (0.15 g, 1 mmol) and 2-methyl-8-hydroxyquinoline (0.16 g, 1 mmol) were loaded into a convection tube; the tube was filled with dry isopropyl alcohol and kept at 333 K. Yellow crystals were collected from the side arm after several days.

### Refinement

Carbon-bound H-atoms were placed in calculated positions [C–H 0.95 to 0.98 Å, *U*_iso_(H) 1.2 to 1.5*U*_eq_(C)] and were included in the refinement in the riding model approximation.

The hydroxy and ammonium H-atoms were located in a difference Fourier map, and were refined with distance restraints of O–H 0.84±0.01 and N–H 0.88±0.01 Å; their temperature factors were refined.

Omitted owing to bad disagreement wwere (5 6 0), (-6 5 3), (-5 6 2), (-4 7 1) and (1 10 3).

### Crystal data

**Table:**

(C_10_H_10_NO)[Sn(C_9_H_6_NO)Cl_4_]	*F*(000) = 1112
*M**_r_* = 564.83	*D*_x_ = 1.830 Mg m^−^^3^
Monoclinic, *P*2_1_/*c*	Mo *K*α radiation, λ = 0.71073 Å
Hall symbol: -P 2ybc	Cell parameters from 6840 reflections
*a* = 8.9431 (3) Å	θ = 2.3–27.5°
*b* = 11.5892 (4) Å	µ = 1.79 mm^−^^1^
*c* = 20.1795 (8) Å	*T* = 100 K
β = 101.347 (4)°	Prism, yellow
*V* = 2050.59 (13) Å^3^	0.30 × 0.25 × 0.20 mm
*Z* = 4	

### Data collection

**Table:**

Agilent SuperNova Dual diffractometer with an Atlas detector	4723 independent reflections
Radiation source: SuperNova (Mo) X-ray Source	4222 reflections with *I* > 2σ(*I*)
Mirror monochromator	*R*_int_ = 0.029
Detector resolution: 10.4041 pixels mm^-1^	θ_max_ = 27.6°, θ_min_ = 2.3°
ω scan	*h* = −11→11
Absorption correction: multi-scan (*CrysAlis PRO*; Agilent, 2012)	*k* = −14→15
*T*_min_ = 0.616, *T*_max_ = 0.717	*l* = −17→26
13674 measured reflections	

### Refinement

**Table:**

Refinement on *F*^2^	Primary atom site location: structure-invariant direct methods
Least-squares matrix: full	Secondary atom site location: difference Fourier map
*R*[*F*^2^ > 2σ(*F*^2^)] = 0.025	Hydrogen site location: inferred from neighbouring sites
*wR*(*F*^2^) = 0.055	H atoms treated by a mixture of independent and constrained refinement
*S* = 1.06	*w* = 1/[σ^2^(*F*_o_^2^) + (0.0193*P*)^2^ + 1.171*P*] where *P* = (*F*_o_^2^ + 2*F*_c_^2^)/3
4723 reflections	(Δ/σ)_max_ = 0.001
262 parameters	Δρ_max_ = 0.48 e Å^−^^3^
2 restraints	Δρ_min_ = −0.54 e Å^−^^3^

### Fractional atomic coordinates and isotropic or equivalent isotropic displacement parameters (Å^2^)

**Table:**

	*x*	*y*	*z*	*U*_iso_*/*U*_eq_	
Sn1	0.252394 (17)	0.454836 (13)	0.684298 (7)	0.01219 (5)	
Cl1	0.06888 (7)	0.32750 (5)	0.71370 (3)	0.01978 (13)	
Cl2	0.36818 (7)	0.51365 (5)	0.79638 (3)	0.01813 (12)	
Cl3	0.43713 (6)	0.30303 (5)	0.68659 (3)	0.01751 (12)	
Cl4	0.07496 (7)	0.61568 (5)	0.67319 (3)	0.01740 (12)	
O1	0.17767 (18)	0.42497 (13)	0.58138 (8)	0.0149 (3)	
O2	0.0249 (2)	0.25623 (14)	0.50577 (9)	0.0221 (4)	
N1	0.4033 (2)	0.56783 (15)	0.63929 (10)	0.0141 (4)	
N2	−0.0535 (2)	0.12664 (17)	0.39515 (10)	0.0161 (4)	
C1	0.5172 (3)	0.6332 (2)	0.66989 (12)	0.0188 (5)	
H1A	0.5461	0.6301	0.7177	0.023*	
C2	0.5963 (3)	0.7065 (2)	0.63406 (13)	0.0224 (6)	
H2A	0.6782	0.7523	0.6572	0.027*	
C3	0.5545 (3)	0.7115 (2)	0.56511 (13)	0.0227 (5)	
H3	0.6070	0.7618	0.5403	0.027*	
C4	0.4339 (3)	0.6427 (2)	0.53060 (12)	0.0186 (5)	
C5	0.3821 (3)	0.6417 (2)	0.45981 (13)	0.0228 (6)	
H5	0.4297	0.6892	0.4316	0.027*	
C6	0.2630 (3)	0.5720 (2)	0.43221 (12)	0.0227 (6)	
H6	0.2276	0.5730	0.3846	0.027*	
C7	0.1908 (3)	0.4985 (2)	0.47190 (12)	0.0187 (5)	
H7	0.1084	0.4511	0.4509	0.022*	
C8	0.2391 (3)	0.4952 (2)	0.54069 (11)	0.0145 (5)	
C9	0.3609 (3)	0.56980 (19)	0.57047 (11)	0.0147 (5)	
C10	−0.2450 (3)	0.1085 (2)	0.29236 (13)	0.0254 (6)	
H10A	−0.2408	0.1929	0.2898	0.038*	
H10B	−0.3371	0.0854	0.3084	0.038*	
H10C	−0.2472	0.0756	0.2474	0.038*	
C11	−0.1082 (3)	0.0656 (2)	0.34005 (12)	0.0178 (5)	
C12	−0.0360 (3)	−0.0387 (2)	0.33034 (12)	0.0197 (5)	
H12	−0.0737	−0.0843	0.2915	0.024*	
C13	0.0878 (3)	−0.0750 (2)	0.37615 (12)	0.0193 (5)	
H13	0.1363	−0.1454	0.3687	0.023*	
C14	0.1453 (3)	−0.0089 (2)	0.43484 (12)	0.0176 (5)	
C15	0.2728 (3)	−0.0407 (2)	0.48462 (13)	0.0215 (5)	
H15	0.3271	−0.1096	0.4797	0.026*	
C16	0.3176 (3)	0.0281 (2)	0.53985 (13)	0.0227 (6)	
H16	0.4045	0.0069	0.5728	0.027*	
C17	0.2382 (3)	0.1294 (2)	0.54906 (12)	0.0198 (5)	
H17	0.2709	0.1751	0.5883	0.024*	
C18	0.1135 (3)	0.1629 (2)	0.50175 (12)	0.0169 (5)	
C19	0.0689 (3)	0.0939 (2)	0.44380 (11)	0.0153 (5)	
H1	0.071 (3)	0.303 (2)	0.5341 (12)	0.036 (9)*	
H2	−0.099 (3)	0.1922 (13)	0.4000 (13)	0.020 (7)*	

### Atomic displacement parameters (Å^2^)

**Table:**

	*U*^11^	*U*^22^	*U*^33^	*U*^12^	*U*^13^	*U*^23^
Sn1	0.01472 (9)	0.01041 (8)	0.01115 (8)	−0.00037 (6)	0.00186 (6)	−0.00024 (6)
Cl1	0.0212 (3)	0.0150 (3)	0.0243 (3)	−0.0044 (2)	0.0074 (2)	0.0002 (2)
Cl2	0.0193 (3)	0.0214 (3)	0.0131 (3)	−0.0001 (2)	0.0016 (2)	−0.0029 (2)
Cl3	0.0196 (3)	0.0159 (3)	0.0163 (3)	0.0045 (2)	0.0019 (2)	−0.0001 (2)
Cl4	0.0208 (3)	0.0137 (3)	0.0176 (3)	0.0038 (2)	0.0036 (2)	0.0006 (2)
O1	0.0173 (8)	0.0144 (8)	0.0127 (8)	−0.0002 (7)	0.0025 (6)	−0.0007 (6)
O2	0.0236 (9)	0.0169 (9)	0.0246 (10)	−0.0012 (8)	0.0018 (8)	−0.0085 (7)
N1	0.0170 (10)	0.0106 (9)	0.0152 (10)	0.0006 (8)	0.0046 (8)	−0.0003 (7)
N2	0.0163 (10)	0.0128 (10)	0.0194 (10)	0.0002 (8)	0.0039 (8)	−0.0004 (8)
C1	0.0201 (12)	0.0167 (12)	0.0197 (12)	−0.0029 (10)	0.0043 (10)	−0.0036 (10)
C2	0.0200 (12)	0.0164 (12)	0.0318 (15)	−0.0059 (10)	0.0075 (11)	−0.0050 (10)
C3	0.0224 (13)	0.0179 (12)	0.0321 (15)	−0.0005 (11)	0.0156 (11)	0.0025 (11)
C4	0.0205 (12)	0.0149 (11)	0.0225 (13)	0.0066 (10)	0.0092 (10)	0.0022 (10)
C5	0.0290 (14)	0.0208 (13)	0.0220 (13)	0.0088 (11)	0.0131 (11)	0.0060 (10)
C6	0.0294 (14)	0.0257 (14)	0.0135 (12)	0.0127 (12)	0.0056 (10)	0.0026 (10)
C7	0.0181 (12)	0.0202 (12)	0.0166 (12)	0.0051 (10)	0.0003 (9)	−0.0013 (10)
C8	0.0159 (11)	0.0118 (10)	0.0162 (11)	0.0050 (9)	0.0041 (9)	0.0008 (9)
C9	0.0168 (11)	0.0120 (11)	0.0158 (11)	0.0061 (9)	0.0047 (9)	0.0017 (9)
C10	0.0208 (13)	0.0287 (15)	0.0239 (14)	−0.0040 (11)	−0.0022 (11)	−0.0005 (11)
C11	0.0174 (12)	0.0183 (12)	0.0181 (12)	−0.0069 (10)	0.0044 (10)	0.0001 (9)
C12	0.0251 (13)	0.0186 (12)	0.0176 (12)	−0.0066 (11)	0.0098 (10)	−0.0050 (9)
C13	0.0238 (13)	0.0143 (11)	0.0219 (13)	−0.0006 (10)	0.0096 (10)	−0.0012 (10)
C14	0.0201 (12)	0.0163 (11)	0.0182 (12)	−0.0028 (10)	0.0083 (10)	0.0014 (9)
C15	0.0211 (13)	0.0201 (13)	0.0243 (13)	0.0026 (11)	0.0066 (11)	0.0048 (10)
C16	0.0194 (12)	0.0278 (14)	0.0198 (13)	−0.0032 (11)	0.0008 (10)	0.0061 (11)
C17	0.0214 (12)	0.0226 (13)	0.0154 (12)	−0.0093 (11)	0.0036 (10)	−0.0012 (10)
C18	0.0183 (12)	0.0144 (11)	0.0188 (12)	−0.0056 (10)	0.0061 (9)	−0.0007 (9)
C19	0.0145 (11)	0.0171 (11)	0.0152 (11)	−0.0047 (10)	0.0052 (9)	0.0016 (9)

### Geometric parameters (Å, º)

**Table:**

Sn1—O1	2.0818 (15)	C6—C7	1.409 (4)
Sn1—N1	2.201 (2)	C6—H6	0.9500
Sn1—Cl1	2.3678 (6)	C7—C8	1.371 (3)
Sn1—Cl2	2.3938 (6)	C7—H7	0.9500
Sn1—Cl3	2.4076 (6)	C8—C9	1.427 (3)
Sn1—Cl4	2.4296 (6)	C10—C11	1.486 (3)
O1—C8	1.348 (3)	C10—H10A	0.9800
O2—C18	1.353 (3)	C10—H10B	0.9800
O2—H1	0.835 (10)	C10—H10C	0.9800
N1—C1	1.321 (3)	C11—C12	1.402 (3)
N1—C9	1.366 (3)	C12—C13	1.362 (3)
N2—C11	1.328 (3)	C12—H12	0.9500
N2—C19	1.372 (3)	C13—C14	1.419 (3)
N2—H2	0.878 (10)	C13—H13	0.9500
C1—C2	1.395 (3)	C14—C19	1.402 (3)
C1—H1A	0.9500	C14—C15	1.412 (3)
C2—C3	1.369 (4)	C15—C16	1.364 (4)
C2—H2A	0.9500	C15—H15	0.9500
C3—C4	1.411 (3)	C16—C17	1.404 (4)
C3—H3	0.9500	C16—H16	0.9500
C4—C9	1.411 (3)	C17—C18	1.373 (3)
C4—C5	1.412 (3)	C17—H17	0.9500
C5—C6	1.365 (4)	C18—C19	1.408 (3)
C5—H5	0.9500		
			
O1—Sn1—N1	77.86 (7)	C8—C7—H7	119.9
O1—Sn1—Cl1	92.52 (5)	C6—C7—H7	119.9
N1—Sn1—Cl1	170.33 (5)	O1—C8—C7	123.2 (2)
O1—Sn1—Cl2	169.80 (5)	O1—C8—C9	118.6 (2)
N1—Sn1—Cl2	91.96 (5)	C7—C8—C9	118.2 (2)
Cl1—Sn1—Cl2	97.65 (2)	N1—C9—C4	121.5 (2)
O1—Sn1—Cl3	88.97 (5)	N1—C9—C8	117.0 (2)
N1—Sn1—Cl3	88.20 (5)	C4—C9—C8	121.5 (2)
Cl1—Sn1—Cl3	92.67 (2)	C11—C10—H10A	109.5
Cl2—Sn1—Cl3	91.28 (2)	C11—C10—H10B	109.5
O1—Sn1—Cl4	87.70 (4)	H10A—C10—H10B	109.5
N1—Sn1—Cl4	87.00 (5)	C11—C10—H10C	109.5
Cl1—Sn1—Cl4	91.66 (2)	H10A—C10—H10C	109.5
Cl2—Sn1—Cl4	91.26 (2)	H10B—C10—H10C	109.5
Cl3—Sn1—Cl4	174.65 (2)	N2—C11—C12	118.2 (2)
C8—O1—Sn1	114.70 (13)	N2—C11—C10	119.0 (2)
C18—O2—H1	110 (2)	C12—C11—C10	122.8 (2)
C1—N1—C9	120.0 (2)	C13—C12—C11	120.5 (2)
C1—N1—Sn1	128.84 (16)	C13—C12—H12	119.8
C9—N1—Sn1	111.09 (14)	C11—C12—H12	119.8
C11—N2—C19	124.2 (2)	C12—C13—C14	120.8 (2)
C11—N2—H2	116.8 (16)	C12—C13—H13	119.6
C19—N2—H2	119.0 (17)	C14—C13—H13	119.6
N1—C1—C2	122.0 (2)	C19—C14—C15	118.4 (2)
N1—C1—H1A	119.0	C19—C14—C13	117.4 (2)
C2—C1—H1A	119.0	C15—C14—C13	124.2 (2)
C3—C2—C1	119.2 (2)	C16—C15—C14	119.7 (2)
C3—C2—H2A	120.4	C16—C15—H15	120.2
C1—C2—H2A	120.4	C14—C15—H15	120.2
C2—C3—C4	120.5 (2)	C15—C16—C17	121.4 (2)
C2—C3—H3	119.8	C15—C16—H16	119.3
C4—C3—H3	119.8	C17—C16—H16	119.3
C3—C4—C9	116.8 (2)	C18—C17—C16	120.5 (2)
C3—C4—C5	124.8 (2)	C18—C17—H17	119.8
C9—C4—C5	118.4 (2)	C16—C17—H17	119.8
C6—C5—C4	119.4 (2)	O2—C18—C17	126.1 (2)
C6—C5—H5	120.3	O2—C18—C19	115.5 (2)
C4—C5—H5	120.3	C17—C18—C19	118.4 (2)
C5—C6—C7	122.2 (2)	N2—C19—C14	118.9 (2)
C5—C6—H6	118.9	N2—C19—C18	119.5 (2)
C7—C6—H6	118.9	C14—C19—C18	121.6 (2)
C8—C7—C6	120.2 (2)		
			
N1—Sn1—O1—C8	−7.95 (15)	C3—C4—C9—N1	1.3 (3)
Cl1—Sn1—O1—C8	171.04 (14)	C5—C4—C9—N1	−178.8 (2)
Cl2—Sn1—O1—C8	−4.8 (4)	C3—C4—C9—C8	−179.0 (2)
Cl3—Sn1—O1—C8	−96.33 (14)	C5—C4—C9—C8	0.9 (3)
Cl4—Sn1—O1—C8	79.48 (14)	O1—C8—C9—N1	−2.5 (3)
O1—Sn1—N1—C1	−176.9 (2)	C7—C8—C9—N1	177.7 (2)
Cl2—Sn1—N1—C1	3.69 (19)	O1—C8—C9—C4	177.8 (2)
Cl3—Sn1—N1—C1	−87.52 (19)	C7—C8—C9—C4	−2.0 (3)
Cl4—Sn1—N1—C1	94.85 (19)	C19—N2—C11—C12	−0.6 (4)
O1—Sn1—N1—C9	6.51 (14)	C19—N2—C11—C10	177.8 (2)
Cl2—Sn1—N1—C9	−172.94 (14)	N2—C11—C12—C13	−0.6 (4)
Cl3—Sn1—N1—C9	95.84 (14)	C10—C11—C12—C13	−179.0 (2)
Cl4—Sn1—N1—C9	−81.79 (14)	C11—C12—C13—C14	0.6 (4)
C9—N1—C1—C2	0.8 (3)	C12—C13—C14—C19	0.6 (4)
Sn1—N1—C1—C2	−175.60 (17)	C12—C13—C14—C15	179.9 (2)
N1—C1—C2—C3	0.3 (4)	C19—C14—C15—C16	0.4 (4)
C1—C2—C3—C4	−0.6 (4)	C13—C14—C15—C16	−178.9 (2)
C2—C3—C4—C9	−0.1 (3)	C14—C15—C16—C17	1.1 (4)
C2—C3—C4—C5	179.9 (2)	C15—C16—C17—C18	−0.9 (4)
C3—C4—C5—C6	−179.4 (2)	C16—C17—C18—O2	177.8 (2)
C9—C4—C5—C6	0.7 (3)	C16—C17—C18—C19	−0.6 (4)
C4—C5—C6—C7	−1.2 (4)	C11—N2—C19—C14	1.9 (4)
C5—C6—C7—C8	0.1 (4)	C11—N2—C19—C18	−177.3 (2)
Sn1—O1—C8—C7	−171.77 (18)	C15—C14—C19—N2	178.8 (2)
Sn1—O1—C8—C9	8.4 (3)	C13—C14—C19—N2	−1.8 (3)
C6—C7—C8—O1	−178.3 (2)	C15—C14—C19—C18	−2.0 (4)
C6—C7—C8—C9	1.5 (3)	C13—C14—C19—C18	177.4 (2)
C1—N1—C9—C4	−1.6 (3)	O2—C18—C19—N2	2.7 (3)
Sn1—N1—C9—C4	175.39 (17)	C17—C18—C19—N2	−178.7 (2)
C1—N1—C9—C8	178.7 (2)	O2—C18—C19—C14	−176.5 (2)
Sn1—N1—C9—C8	−4.3 (2)	C17—C18—C19—C14	2.1 (4)

### Hydrogen-bond geometry (Å, º)

**Table:**

*D*—H···*A*	*D*—H	H···*A*	*D*···*A*	*D*—H···*A*
O2—H1···O1	0.84 (1)	1.86 (1)	2.683 (2)	168 (3)
N2—H2···O2	0.88 (1)	2.33 (2)	2.667 (3)	103 (2)
